# A Novel Proteomic Analysis of the Modifications Induced by High Hydrostatic Pressure on Hazelnut Water-Soluble Proteins

**DOI:** 10.3390/foods3020279

**Published:** 2014-05-05

**Authors:** Nuria Prieto, Carmen Burbano, Elisa Iniesto, Julia Rodríguez, Beatriz Cabanillas, Jesus F. Crespo, Mercedes M. Pedrosa, Mercedes Muzquiz, Juan Carlos del Pozo, Rosario Linacero, Carmen Cuadrado

**Affiliations:** 1 Genetics Department, Biology Faculty, Complutense University of Madrid, Madrid 28040, Spain; E-Mails: nuph_seth@hotmail.com (N.P); e.iniesto@gmail.coM (E.I.); charolin@ucm.es (R.L.); 2 Food Technology Department, National Institute for Agricultural and Food Research and Technology (INIA), Ctra. La Coruña Km. 7.5, Madrid 28040, Spain; E-Mails: burbano@inia.es (C.B.); mmartin@inia.es (M.M.P.); muzquiz@inia.es (M.M.); 3 Allergy Service, Research Institute Hospital 12 de Octubre (i+12), Avenida de Córdoba s/n, Madrid 28041, Spain; E-Mails: juliarodriguez2@telefonica.net (J.R.); Beatriz.Cabanillas@ukb.uni-bonn.de (B.C.); jfcrespo@isciii.es (J.F.C.); 4 Center for Biotechnology and Plant Genomic, Polytechnic University of Madrid-National Institute for Agricultural and Food Research and Technology (UPM-INIA), Montegancedo Campus, Boadilla del Monte, Madrid 28660, Spain; E-Mail: jc.delpozo@upm.es

**Keywords:** hazelnut, high hydrostatic pressure, immunoreactivity, ProteomeLab PF-2D

## Abstract

Food allergies to hazelnut represent an important health problem in industrialized countries because of their high prevalence and severity. Food allergenicity can be changed by several processing procedures since food proteins may undergo modifications which could alter immunoreactivity. High-hydrostatic pressure (HHP) is an emerging processing technology used to develop novel and high-quality foods. The effect of HHP on allergenicity is currently being investigated through changes in protein structure. Our aim is to evaluate the effect of HHP on the protein profile of hazelnut immunoreactive extracts by comparative proteomic analysis with ProteomeLab PF-2D liquid chromatography and mass spectrometry. This protein fractionation method resolves proteins by isoelectric point and hydrophobicity in the first and second dimension, respectively. Second dimension chromatogram analyses show that some protein peaks present in unpressurized hazelnut must be unsolubilized and are not present in HHP-treated hazelnut extracts. Our results show that HHP treatment at low temperature induced marked changes on hazelnut water-soluble protein profile.

## 1. Introduction

High-hydrostatic pressure (HHP) is considered an emerging processing technology used to develop novel and high-quality foods. This novel-processing technique even renders harmless foods which would be of considerable benefit to consumers. HHP treatment of foods can be used to create new products (new texture or taste) or to obtain analogue products with minimal effect on flavor, color and nutritional value and without any thermal degradation. It is well established that higher pressure has a disruptive effect on the tertiary and quaternary structure of most globular proteins, with relatively little influence on the secondary structure. Therefore, higher hydrostatic pressure can unfold proteins. The typical pressure needed for the unfolding is around 500 MPa but it varies from protein to protein, in the range from 100 MPa to 1 GPa or reaching even higher pressures in special cases. The effect of HHP on immunoreactive proteins is being currently investigated through changes in protein structure [1]. Such effects have been studied in: beef [2,3], apple [4,5], celery [4] and in nuts such as peanut [5]. However, there is scarce information on the effects of such food processing techniques on hazelnut (*Corylus avellana* L.) immunoreactive proteins.

Food allergies to hazelnut represent an important health problem in industrialized countries because of their high prevalence and severity [6]. Several hazelnut allergens are well characterized being Cor a 1 (18 KDa, Bet v 1 family) the major one. Other allergenic proteins are Cor a 2 (profilin), Cor a 8 (lipid transfer protein, LPT), Cor a 9 (11S globulin), Cor a 11 (vicilin-like protein) and Cor a 12, Cor a 13 and Cor a 14 belonging to the 2S albumins [7].Understanding how food processing affects the allergenic proteins could be important to control food allergenicity risk.

Bioinformatics tools for database searching enable the quick identification of sequences of interest. Tools for sequence comparison, motif searching or sequence profiling assist researchers to identify biologically relevant sequences similarities. The guidelines to assess potential allergenicity of proteins using bioinformatics in a step-by-step procedure are well established [8,9].

Plant comparative proteomics is becoming increasingly attractive as the rapidly expanding plant genomic and Expressed Sequence Tags (EST) databases provide new opportunities for protein identification. A partial automation of this procedure consisting of a robotic lift of protein spots embedded in the gel, followed by extraction, destaining and protein digestion, has been finalized with reasonable success to the further protein characterization and identification by mass spectrometry (MS) [10]. Proteome analyses have also been performed in a “gel free” condition by using protein fractionation procedures based entirely on liquid chromatography [11,12]. ProteomeLab PF-2D introduced a two dimensional liquid chromatography based on a high-performance chromatofocusing in the first dimension followed by high-resolution reversed-phase chromatography in the second dimension [13,14]. The ProteomeLab PF-2D has become available for sample fractionation and was more resolutive at extreme pHs, both acid and basic. It offers automation of the fractionation processes and resolves proteins by isoelectric point and hydrophobicity in the first and second dimension, respectively [15].

In this study, we have undertaken a comparative proteomic analysis of the effect of HHP at low temperature on water-soluble protein profile of hazelnut immunoreactive extracts using a novel proteomic approach that combines ProteomeLab PF-2D liquid chromatography and mass spectrometry analysis.

## 2. Experimental Section

### 2.1. Samples and High Hydrostatic Pressure (HHP) Treatments

The hazelnut (*Corylus avellana* L.) var Negreta used in this work was provided by the hazelnut collection of Institut de Recerca i Tecnología Agroalimentàries (IRTA-Mas de Bover, Tarragona, Spain) [16].

**Figure 1:**
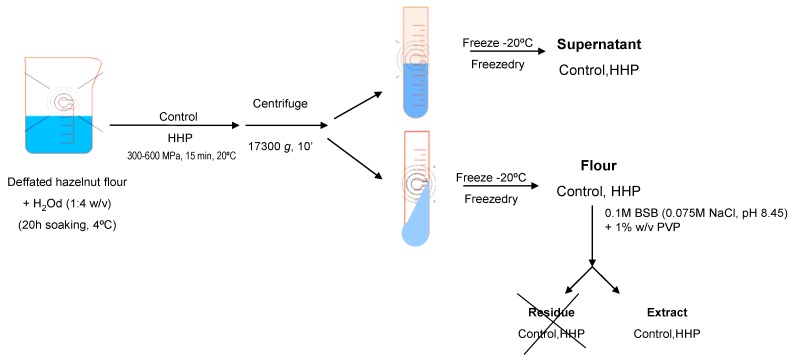
Scheme of high hydrostatic pressure (HHP) treatment of hazelnut samples.

Hazelnuts were ground and defatted with *n*-hexane (34 mL/g of flour) for 4 h and air-dried after filtration of the *n*-hexane. High-pressure experiments conditions were carried out according to Omi *et al.* [17] and Kato *et al*. [18]. Hazelnut defatted flours were dissolved in distilled water (1:4 w/v) 20 h before HHP treatment. The flours were subjected to HHP, using pressures of 300, 400, 500 and 600 MPa for 15 min in a multivessel high-pressure equipment (HHP, ACB, France) at 20 °C (Figure 1).

### 2.2. Protein Electrophoresis and Immunoblotting

1D SDS-PAGE was performed according to Laemmli [19]. Protein extracts of hazelnut unpressurized (control) and HHP-treated (300–600 MPa) of supernatant and flour were mixed with XT Sample Loading Buffer (Bio-Rad, Hercules, CA, USA) heated at 95 °C for 5 min, electrophoresed in 12% Bis-Tris precast gel (Bio-Rad, Hercules, CA, USA). Proteins were visualized with Coomassie brilliant blue R250 staining.

Western blotting was performed by electrophoretic transfer to polyvinylidene difluoride (PVDF) membranes at 250 mA for 100 min, at room temperature, essentially according to the method of Towbin *et al.* [20]. After blocking with 5% bovine serum albumin (BSA) (w/v) in phosphate buffered saline (PBS), membranes were incubated overnight with the serum pool from fifteen patients sensitized to hazelnut (serum specific IgE > 0.35 kilounits/L quantified by the CAP-FEIA assay) (1:10 dilution), washed, and then treated with mouse anti-human IgE mAb HE-2 ascitic fluid (1:3000 dilution for 2 h) [21]. After washing, a rabbit anti-mouse IgG peroxidase-conjugated antibody (1:5000 dilution for 1 h; DAKO, Glostrup, Denmark) was added. Detection of IgE-binding components was achieved by means of enhanced chemiluminescence, according to the manufacturer’s instructions (Amersham Biosciences, Little Chalfont, UK).

### 2.3. Protein Sample Preparations and 2D-Liquid Chromatography Analysis

Total protein extracts of unpressurized and HHP-treated hazelnut previously tested for IgE-reactivity were used for comparative proteomic analysis with ProteomeLab PF-2D liquid chromatography (Beckman Coulter, Fullerton, CA, USA).

Unpressurized and HHP-treated hazelnut proteins were extracted with 0.1 M borate saline buffer (BSB, 0.075 M NaCl, pH 8.5) plus 1% (w/v) PVP at a 1:10 w/v ratio for 1 h at 4 °C by stirring. The extract was clarified by centrifugation at 27,000× *g* for 30 min at 4 °C, and the supernatants were dialyzed against distilled H_2_O for 48 h at 4 °C using a dialysis membrane with a cut-off of 3.5 kDa and freeze-dried. The protein content of each sample was measured by the Bradford dye binding assay (Bio-Rad, Hercules, CA, USA) using bovine serum albumin (Sigma, St. Louis, MO, USA) as a standard. The nitrogen contents of the samples were determined by LECO analysis according to standard procedures based on Dumas method [22]. The total protein content was calculated as N × 5.3 [22]. The analyses were carried out in duplicates.

Protein extracts (2.5 mg of protein) from unpressurized and HHP-treated hazelnut were subjected to 2-D LC analysis using a ProteomeLab PF-2D instrument (Beckman-Coulter, Fullerton, CA, USA) and the protocol recommended by the manufacturer (ProteoSep^®^, Chemistry Kit, Sigma, St. Louis, MO, USA). The first-dimension separation was carried out by chromatofocusing on a High Performance ChromatoFocusing (HPCF) 1-D column (250 mm × 2.1 mm internal diameter, 300 Å pore size). The column was equilibrated at pH 8.5 with CF Start Buffer for 250 min at 0.2 mL/min. The pH gradient began after 20 min of sample injection when the CF Eluent Buffer at pH 4.0 moved through the column, gradually decreasing the pH from 8.5 to 4.0. Proteins were eluted according to their isoelectric points (pI) and, in the final step, the most acidic ones were eluted with 1 mol NaCl, 0.2% *n*-octylglucoside. All fractions were collected in 96 well plates using an automated collector.

All the different pH fractions collected from the first dimension were resolved on a reverse phase C18 column (HPRP column: 4.66 mm × 3.3 mm, 1.5 μm particle size,). Of each fraction, 200 μL was run through the column in solvent A (0.1% v/v TFA in water), and the proteins were then eluted with a linear gradient (0%–100%) of solvent B (0.08% v/v trifluoroacetic acid in acetonitrile) for 35 min. Separation was performed at 0.75 mL/min, and the temperature column was maintained at 50 °C. Eluted proteins were monitored by ultraviolet light at 214 nm of absorbance. The different fractions of the first dimension were collected in 12 plates (each 96 well) using an automated collector. All CF profiles were elaborated and compared using 32 Karat V1.01 software (Beckman-Coulter, Fullerton, CA, USA). Quantitative analysis of unpressurized and HHP-treated hazelnut protein peak areas and heights were performed using the Mapping tools software V1.0 (Beckman-Coulter, Fullerton, CA, USA).

Fractions from the second dimension were analyzed by 1D SDS-PAGE as above described and selected bands were manually excised for protein identification by mass spectrometry and database search.

### 2.4. Protein Identification by MS and Data Base Search

Immunoreactive hazelnut proteins modified by high hydrostatic pressure were analyzed by MS and data base search in order to determine their identification. Proteins were in-gel digested with trypsin (Sequencing Grade Modified, Promega, Madrid, Spain) in the automatic Investigator ProGest robot of Genomic Solutions. Briefly, excised gel bands were washed sequentially with 50 mM ammonium bicarbonate (NH_4_HCO_3_) buffer and acetonitrile. Eluted fractions were evaporated to a final volume of 10 μL. Protein digestions were carried out by incubating the samples in 50 mmol/L NH_4_HCO_3_ and 10 mmol/L dithiothreitol at 60 °C for 1 h. The alkylation of the reduced sulfhydryl groups was performed by adding 55 mmol/L iodoacetamide at 25 °C for 30 min in the dark. Proteins were digested by adding 1.5 μL of trypsin (125 μg/mL) and incubating at 37 °C overnight. The reaction was stopped with 1% of formic acid. Tryptic peptides were desalted and concentrated with ZipTipC18 columns according to the manufacturer’s recommendation. Peptides were eluted in 0.1% trifluoroacetic acid , 50% acetonitrile for matrix-assisted laser desorption ionization time-of-flight (MALDI-TOF)-MS analysis, and with 1% formic acid, 50% methanol for electrospray MS analysis. To increase salt removal, samples were washed with 3–5 cycles of 0.1% triflouroacetic acid as wash solution. The solution was spotted directly onto a MALDI target and analyzed by MALDI-TOF/TOF off-line coupled LC/MALDI-MS/MS. MS analyses were performed automatically with a 4700 Analyzer MALDI-TOF/TOF instrument (Applied Biosystems, Carlsbad, CA, USA). First, MS spectra of all spotted fractions were acquired in the positive reflector mode for peak selection (S/N > 20, excluded precursor with 200 resolution), and further MS/MS spectra acquisition was done using the Collision Induced Dissociation of selected peaks. The search of filtered peptides was performed in batch mode using GPS Explorer V 3.5.0 software with a licensed version of MASCOT, in the Swiss-Prot Database. The MASCOT search parameters were: (1) species, *Coryllus avellana*; (2) allowed number of missed cleavages (only for trypsin digestion), 1; (3) considered modifications, cysteine as carboamidomethyl derivate and methionine as oxidized methionine; (4) peptide tolerance, ±50 ppm; (5) MS/MS tolerance, ±0.3 Da; and (6) peptide charge, +1 [23].

## 3. Results and Discussion

3.1. 1D Analysis and Immunoblottting

**Figure 2:**
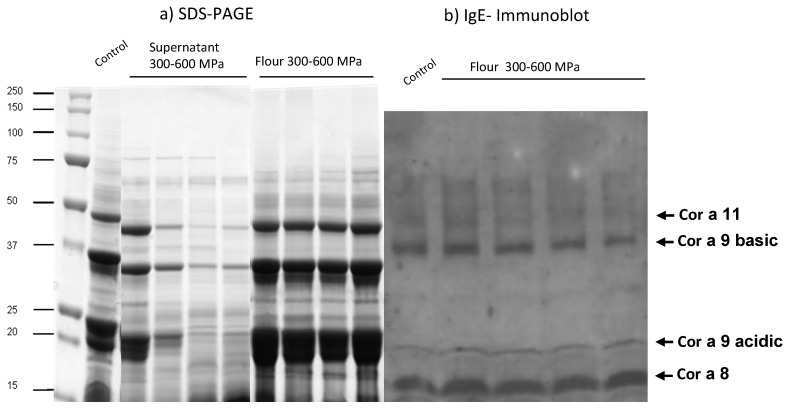
(**a**) SDS-PAGE patterns of supernatant and flour protein extract from control and processed hazelnuts samples and (**b**) IgE immunoblot analysis of control and processed hazelnut samples with a serum pool from subjects with specific IgE to hazelnut (31.3 kU/L).

The protein migration patterns of hazelnut before (control) and after high-pressure treatments are shown by SDS-PAGE (Figure 2a). Samples were also analyzed for differences in IgE binding by immunoblot using pool sera from 15 patients sensitized to hazelnut (Figure 2b). Defatted flours that were directly solubilized in sample buffer were used for the immunoassays carried out in this study. The results showed that electrophoretic migration patterns of high-pressure treated flour hazelnut proteins were similar to the control hazelnut.

However, the band intensity of supernatants was diminishing according the pressure increase from 300 to 600 MPa (Figure 2a). The possibility that HHP resulted in variable aggregation depending on the applied pressure could explain these findings. This is in agreement with Somkuti and Smeller [1].

The IgE-immunoblot patterns are similar in control and pressure hazelnuts treated samples from 300 to 600 MPa, showing multiple immunoreactive proteins (Figure 2b). In these samples, most immunoreactive proteins fall in the central and lower area of the gel covering 15–60 kDa. The IgE immunoblotting showed that IgE of pool sera from sensitized to hazelnut recognized bands in all the samples at 50 kDa, 40 kDa, 20 kDa and 9 kDa which might correlate with Cor a 11, Cor a 9, Cor a 1 and Cor a 8 allergens [7].

### 3.2. Hazelnut Proteins Analyzed by Liquid Chromatography

**Figure 3:**
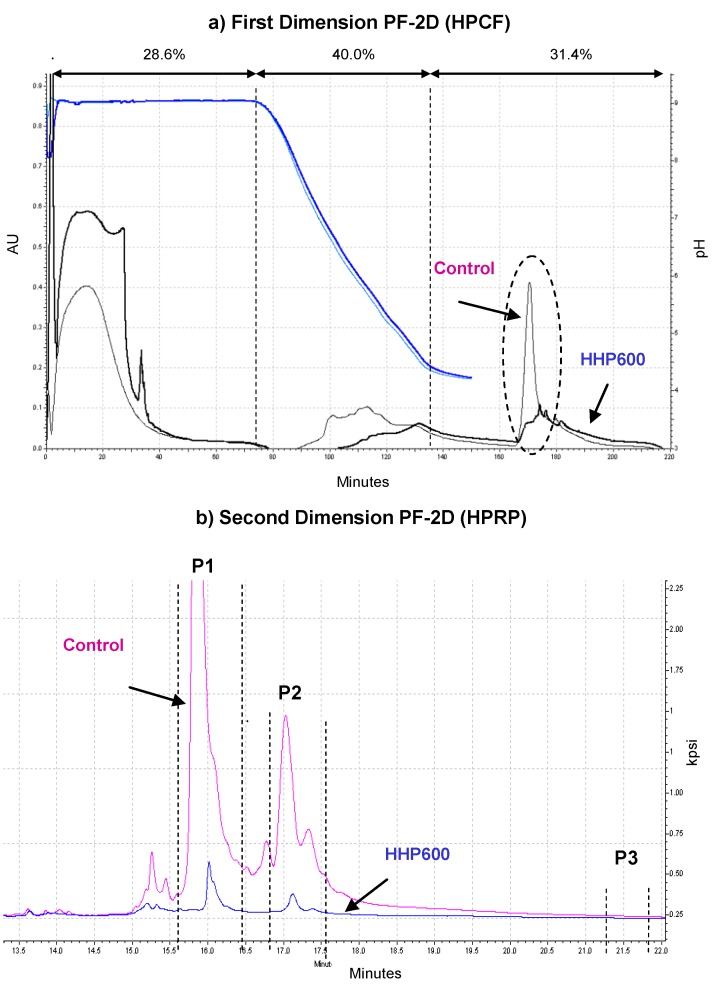
Chromatographic analysis of hazelnut proteins using ProteomeLab PF-2D: (**a**) Chromatofocusing of total proteins separated by pH gradient 8.0–4.0. Fraction number 29 of control and 34 of HHP hazelnut were fractionated in the second dimension. (**b**) High performance reverse phase chromatography, fractions are separated by hydrophobicity. Proteins contained in peaks marked P1, P2 and P3 were separated by SDS-PAGE for subsequent MS analyses to determine their identity.

In the ProteomeLab PF-2D system, 2.5 mg of untreated (control) and HHP (600 MPa)-treated hazelnut protein extracts were injected into the HPCF column and were recovered as follow: 40.0% in the 8.0–4.0 pH gradient, 28.6% before the initiation of the pH gradient, and the remaining 31.4% eluted as different peaks when the column was finally washed with high salinity buffer (Figure 3a). The fractions showing higher differences between untreated and HHP treated hazelnut were collected. We collected seven fractions in the control sample and 10 fractions in de HHP-treated hazelnut sample with high salinity buffer and they were subjected to a second dimension using the high performance reverse-phase chromatography column (Figure 3b).

The protein profiles obtained showed important differences between the two samples (control and HHP 600 samples) with much lesser and smaller peaks in HHP 600 hazelnut. The fractions collected after the second dimension were concentrated in three ones, namely P1 (from 23 to 26), P2 (from 27 to 29) and P3 (from 34 to 35) in control as well as HHP treated hazelnut.

### 3.3. Proteins Identified in Raw and HHP Hazelnuts Samples

**Figure 4:**
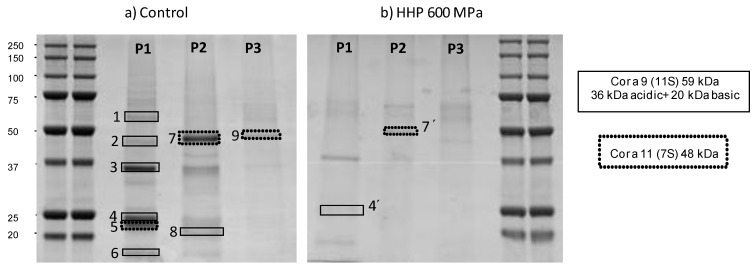
SDS-PAGE of the different fractions collected and concentrated (P1, P2 and P3) from the second dimension chromatography column (HPRP) of control hazelnut **(a)** and HHP 600 MPa hazelnut **(b)**. Proteins were visualized by Coomassie Blue. The rectangles indicate the protein bands that were identified by MS analysis.

P1, P2 and P3 fractions from raw hazelnuts (control) and HHP600 hazelnut samples were further analyzed by 1D SDS-PAGE (Figure 4a,b). Control and HHP 600 hazelnut had a distinct protein band pattern, in band number as well as band intensity, in agreement with the differences showed in the second dimension absorbance profile between both hazelnut samples (Figure 3b).

Finally, nine of these polypeptide bands of control hazelnut and two bands in HHP 600 hazelnut were manually excised for MALDI-TOF-TOF and LC-ESI-MS/MS identification. Table 1 summarizes the polypeptide identification data of raw hazelnut sample. The major bands were tryptic digested in order to carry out the analysis by MALDI-TOF/MS. A peptide mass fingerprint search allowed us to identify the bands 1, 2, 3, 4, 6 and 8 of raw hazelnut as a 11S globulin-like of *C. avellana* (59 kDa) (Table 1). The bands 5, 7 and 9 of raw hazelnut were identified as a 7S vicilin-like of *C.* *avellana* (48 kDa). The identification carried out in HHP600 treated sample (Table 2) showed only one band (4′) corresponding to the 11S globulin-like and another one (7′) identified as the 7S vicilin-like. According to the hazelnut allergen description [7], the bands identified as 11S globulin-like could correspond to the putative Cor a 9 allergen (59 kDa) which is composed of a 36 kDa acid subunit and a 20 kDa basic subunit. The 7S vicilin-like bands could correlate with the Cor a 11 allergen (48 kDa). 

Proteome analysis is a tool that can be used both to visualize and compare complex mixtures of proteins and to gain a large amount of information about the individual proteins involved in specific biological responses. In this work, proteomic analysis by bidimensional chromatography (PF-2D) shows that the high hydrostatic pressure (HHP) induces significant changes in the proteome of hazelnut extracts in agreement with results on differential solubility after HHP process reported by other authors in several foods [1,4,18,24]. Our results show that the protein solubility became different among the fractions from 300 to 600 MPa. The identification by MALDI-TOF/MS of the proteins affected by high pressure indicated that among the proteins which are insolubilized by high pressure, there are some legumins and vicilins that could correspond to the allergen Cor 9 (11S) and Cor 11 (7S) according to data base search. 

**Table 1:** Proteins separated by ProteomeLab PF-2D from raw hazelnuts and identified by MALDI-TOF/MS.

Band No.	No. access	Protein identification	Mascot score *	Mass (Da)	Matched peptides
1	gi|18479082	11S globulin-like protein (*C. avellana*)	73	59,605	12
2	gi|18479082	11S globulin-like protein (*C. avellana*)	161	59,605	19
3	gi|18479082	11S globulin-like protein (*C. avellana*)	136	59,605	6
4	gi|18479082	11S globulin-like protein (*C. avellana*)	187	59,605	20
5	gi|19338630	48-kDa glycoprotein precursor (*C. avellana*)	199	51,110	21
6	gi|18479082	11S globulin-like protein (*C. avellana*)	218	59,605	13
7	gi|19338630	48-kDa glycoprotein precursor (*C. avellana*)	256	51,110	24
8	gi|18479082	11S globulin-like protein (*C. avellana*)	65	59,605	11
9	gi|19338630	48-kDa glycoprotein precursor (*C. avellana*)	199	51,110	21

* Protein scores greater than 42 are significant (*p* < 0.05). Protein score is −10 × Log (*P*), where *P* is the probability that the observed match is a random event.

**Table 2:** Proteins separated by ProteomeLab PF-2D from hazelnuts HHP 600 MPa and identified by MALDI-TOF/MS.

Band No.	No. access	Protein identification	Mascot score *	Mass (Da)	Matched peptides
4′	gi|18479082	11S globulin-like protein (*C. avellana*)	205	59,605	10
7′	gi|19338630	48-kDa glycoprotein precursor (*C. avellana*)	194	51,110	21

* Protein scores greater than 42 are significant (*p* < 0.05). Protein score is −10 × Log (*P*), where *P* is the probability that the observed match is a random event.

## 4. Conclusions

The proteomic approach described here allows a deeper and more detailed study of the modifications induced by high pressure. The HHP treatment employed in this study is not effective to alter the immunoreactivity to hazelnut proteins but the protein solubility became different after HHP processing. Although at present there is not an accepted method to reduce the allergenicity of foods, a combination of treatments including high hydrostatic pressure with others, such as protease digestion, could be a successful strategy towards hypoallergenization. To reach this, we have to collect more information about the pressure behavior of these proteins at various environmental conditions and in presence of food additives. There is a definite need for further studies at basic scientific level, which could report the effect of pressure on the 3D structure of the allergenic proteins. The complexity of food processing demonstrates the importance of understanding its impact at the molecular level if risk assessors are to move towards knowledge-based ways of managing allergen risks.
